# An Unusual Case of Foreign Body in the Anterior Abdominal Wall

**DOI:** 10.4021/jocmr2009.11.1271

**Published:** 2010-04-15

**Authors:** Kim Weng Chan

**Affiliations:** Department of General Surgery, Belfast City Hospital, Belfast BT9 7AB, United Kingdom. E-mail: kim.chan@nhs.net

## Abstract

Accidental or intentional ingestion of sharp metallic foreign bodies is a common clinical occurrence. Predisposing factors include excessive alcohol intake, mental impairment and psychiatric illnesses. Usually, the detection of a migratory foreign body is incidental when patients present with unrelated symptoms. A 50-year-old woman attended the surgical outpatient clinic with a foreign body in the anterior abdominal wall. A foreign body was easily palpable on the right upper quadrant of abdomen. Patient was admitted electively and taken to theatre for removal of foreign body under general anaesthesia. Patient made an uneventful recovery and was discharged with no further follow-up.

**Keywords:**

Foreign body; Abdomen; Ingestion; Perforation

## Introduction

Accidental or intentional ingestion of sharp metallic foreign bodies is a common clinical occurrence. Predisposing factors include excessive alcohol intake, mental impairment and psychiatric illnesses. Most swallowed foreign bodies are excreted in the faeces without causing any major disturbances to the gastrointestinal tract [1]. Migration of such foreign bodies after perforating the bowel is rare and is usually silent [2]. Although these foreign bodies may migrate to almost any intra-abdominal organ, migration to the liver, mesentery or abdominal wall is extremely rare [3]. Usually, the detection of a migratory foreign body is incidental when patients present with unrelated symptoms. We report such a case of sharp foreign body found within the anterior abdominal wall.

## Case Report

A 50-year-old woman attended the surgical outpatient clinic with a foreign body in the anterior abdominal wall. She was referred by the physician who had initially admitted her to the medical ward with deliberate self-poisoning in 2005. She denied ingesting foreign body apart from her usual medications. Her past medical history includes deliberate self-harm, taking overdose of medications, bulimia nervosa, anxiety and alcohol excess. She has no past surgical history of note apart from tonsillectomy.

On further questioning, she denied ingesting or inserting any foreign object into her abdomen. She alleged that she was raped anally once prior to 2005 but denied that the assailant had inserted any foreign object into her anal canal. She also denied swallowing any foreign object in the past. She has no pain or other symptoms referable to this object. On examination, there was no scar visible in the abdominal wall. A foreign body is easily palpable in the right upper quadrant of the abdominal wall.

**Figure 1.**
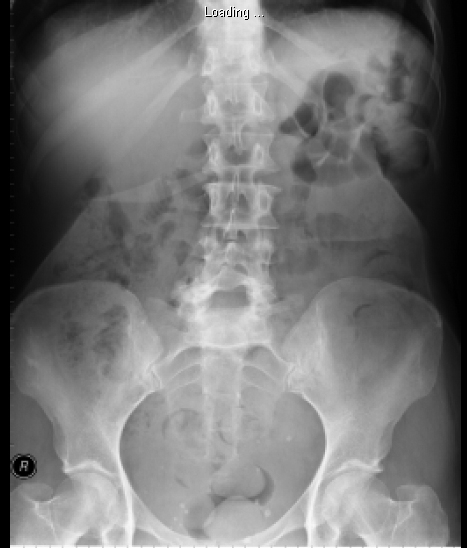
Plain abdominal x-ray showing the foreign body in the right upper quadrant of abdomen.

A plain abdominal X-ray confirmed the presence of a diamond shaped foreign body in the right upper quadrant of the abdomen (Fig. 1). Computed tomography (CT) scan of the abdomen was performed to assess the foreign object. The CT showed a radio-dense foreign body measuring 65 × 14 × 4 mm deep to subcutaneous fat of anterior abdominal wall on the right side, lying just outside rectus abdominis. The CT also confirmed no component of foreign body is seen to enter the peritoneum.

She was asymptomatic in relation to this foreign body. She was then follow-up in the surgical outpatient clinic for consideration of removal of the foreign body if it causes any symptoms. However, she failed to attend the surgical outpatient on several occasions. She was referred back to the surgical outpatient clinic 2 years later by her general practitioner as the foreign body is causing her discomfort.

An ultrasonography was arranged to further evaluate the foreign body. The ultrasonography confirmed the presence of foreign body as described in the previous CT scan, which lies in the subcutaneous tissue in the right hypochondrium, approximately 2.5 cm below the skin surface. She was keen to have this removed.

**Figure 2.**
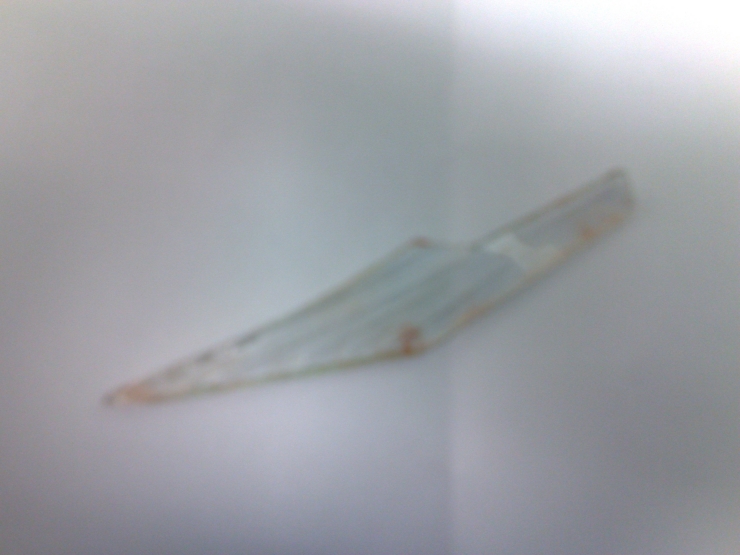
Arrow-shaped foreign body removed from the anterior abdominal wall.

She was admitted electively and taken to theatre for removal of the foreign body under general anaesthesia. The foreign body was found underneath the subcutaneous tissue of the abdominal wall. This foreign body turned out to be an arrow shaped glass (Fig. 2). Patient made an uneventful recovery and was discharged home with no further follow-up.

## Discussion

Case reporting an ingested sharp metallic body perforating the gut, though presenting in the literature are surprisingly rare [3]. Perforation and migration of such a foreign body may be silent. Patients may present with unrelated symptoms and the discovery of foreign body on radiological examination of the abdomen may come as a surprise [3]. History of introduction of foreign body is usually difficult to obtain. Such intra-abdominal foreign body can lead to insidious unrelated presentation or more catastrophic events including perforation or obstruction of the bowel, migration to almost any intra abdominal or rarely to even extra abdominal sites. Migration to the liver, mesentery or the anterior abdominal wall, however is extremely rare [4-6].

Variable time periods ranging from months to years have been reported between the introduction of a foreign body and the occurrence of symptoms. Patient is asymptomatic as in this case. She denied ingesting or inserting a foreign body. Abdominal examination did not reveal any penetrating wound or scar. Besides, CT also confirmed that no component of foreign body was seen to enter the peritoneum. The clinical history and radiological investigations made this an interesting and unusual case as no explanation could be offered for the presence of the sharp foreign body in the anterior abdominal wall. The possibility could be silent gastrointestinal perforation with migration of the foreign body to the anterior abdominal wall after patient ingested it.

Metals and glass fragments are easily seen on plain x-ray while plastic and wood are radiolucent and are only seen by CT scan or ultrasonography [7, 8]. A plain radiograph of a sample piece of suspected foreign body, if available, is quite useful. Radiograph with the point of entry marked by a radiopaque marker and immediate preoperative radiograph should also be taken to decide the location and trajectory of removal. This is a useful exercise because certain foreign bodies may migrate or even embolise to distant sites [7]. Ultrasonography has a problem solving and a corroborative role in foreign body removal [8]. The possibility of penetrating-migrating by a sharp foreign body is widely accepted and the whole process of disease entity is mostly symptom-free and usually an event of time-spending.

In conclusion, ingestion of foreign bodies rarely causes perforation of bowel and most of these will pass spontaneously or it may migrate silently to other parts of the body. Surgical intervention should be offered to patient if the foreign body causes any symptoms.
